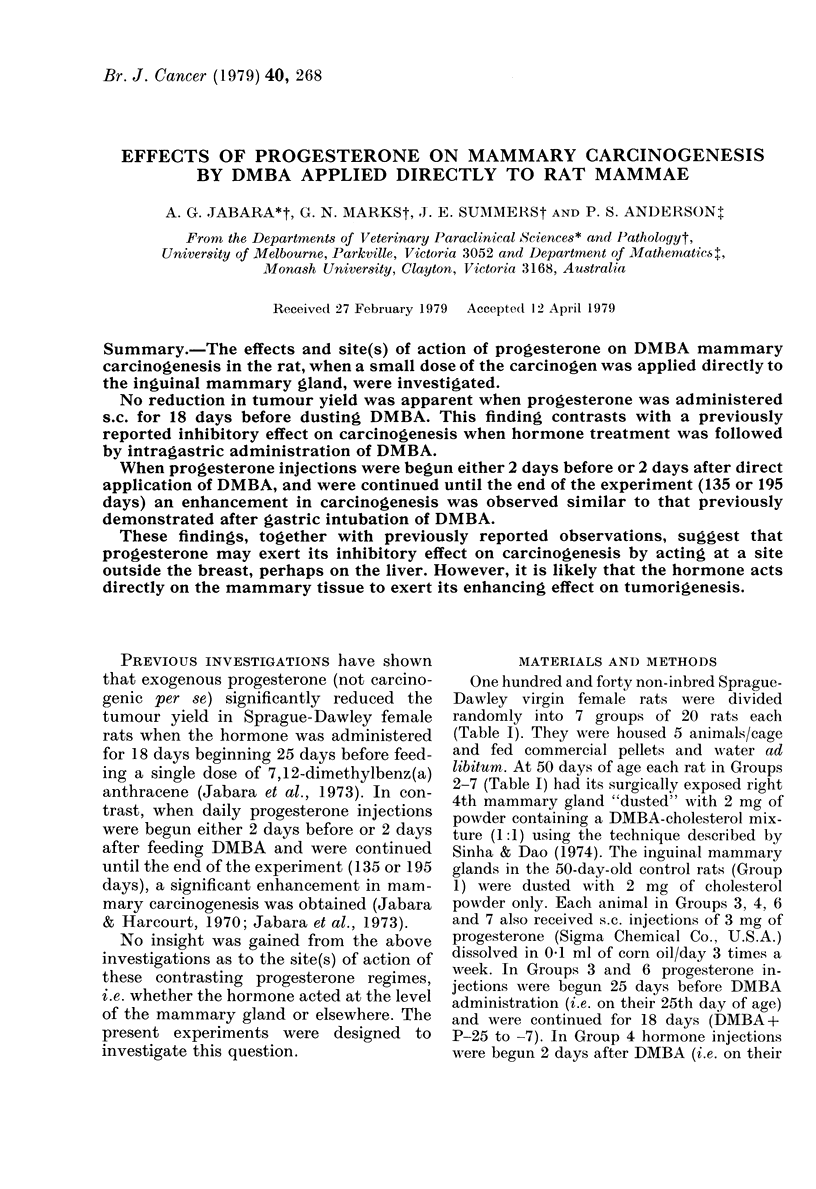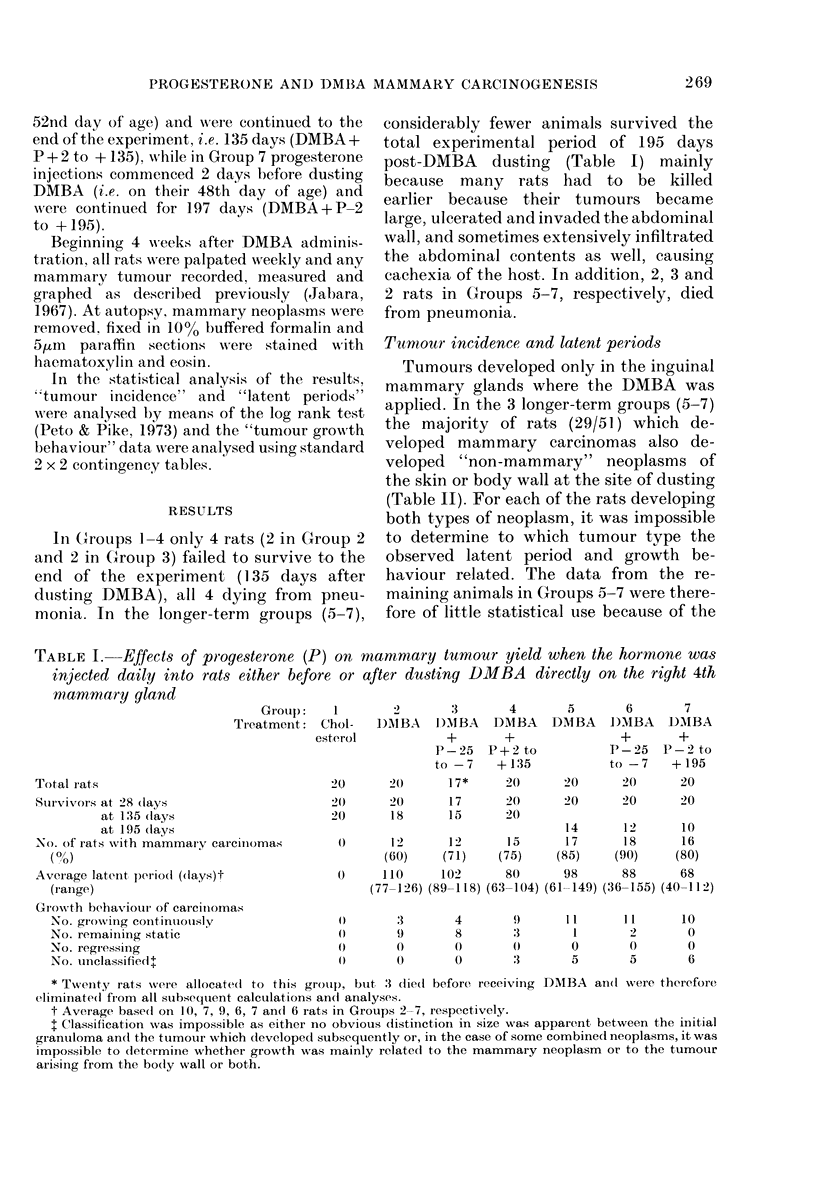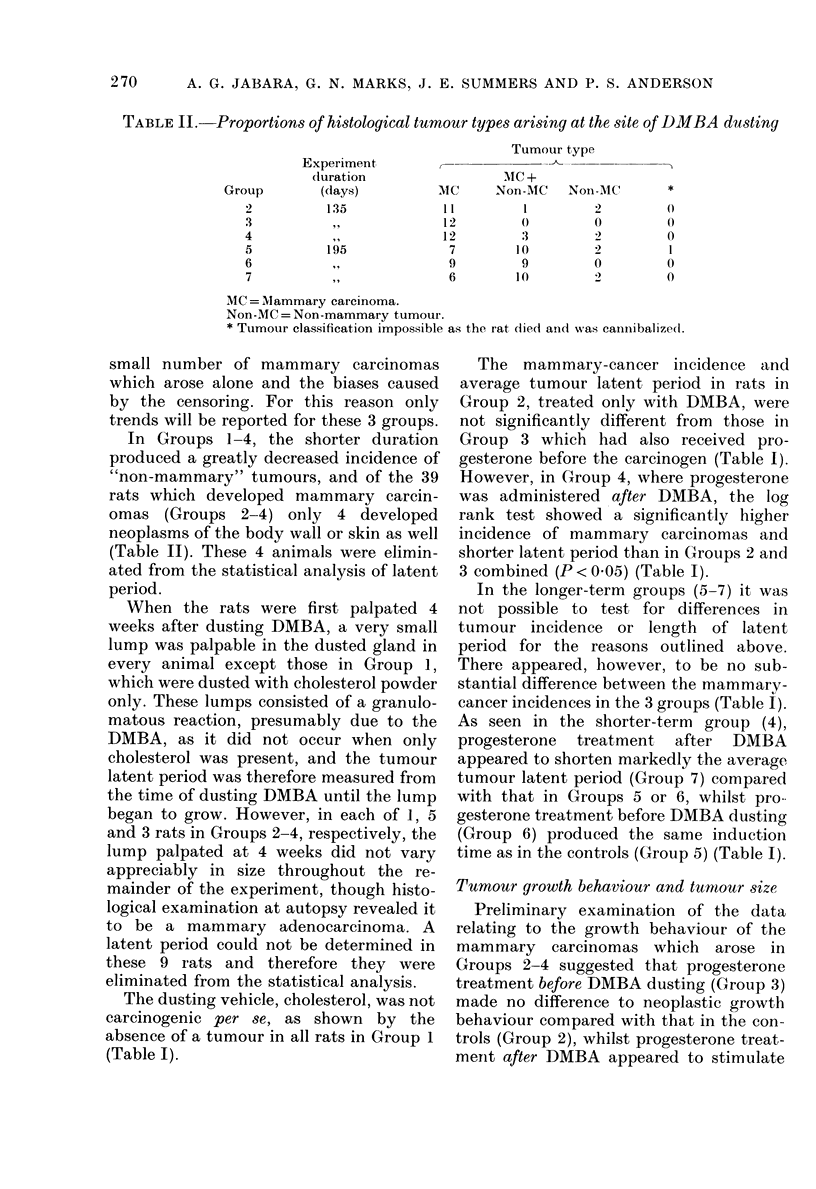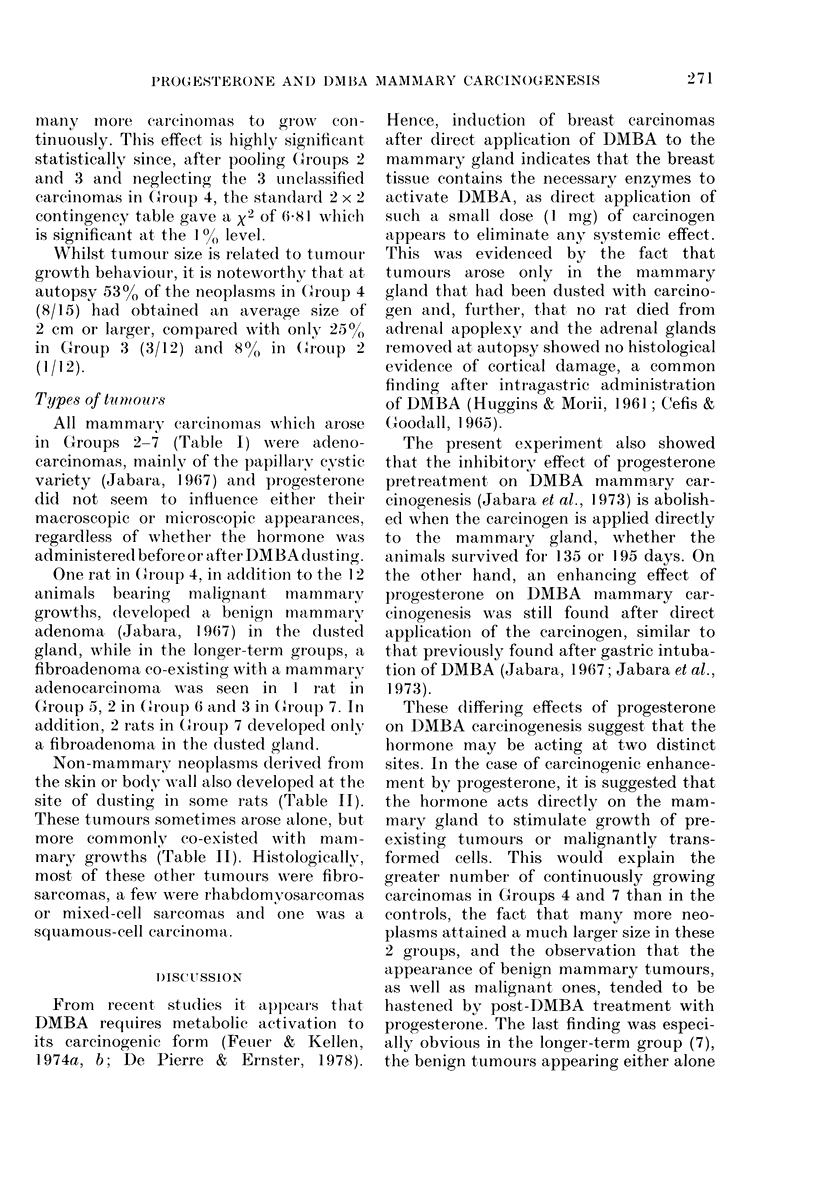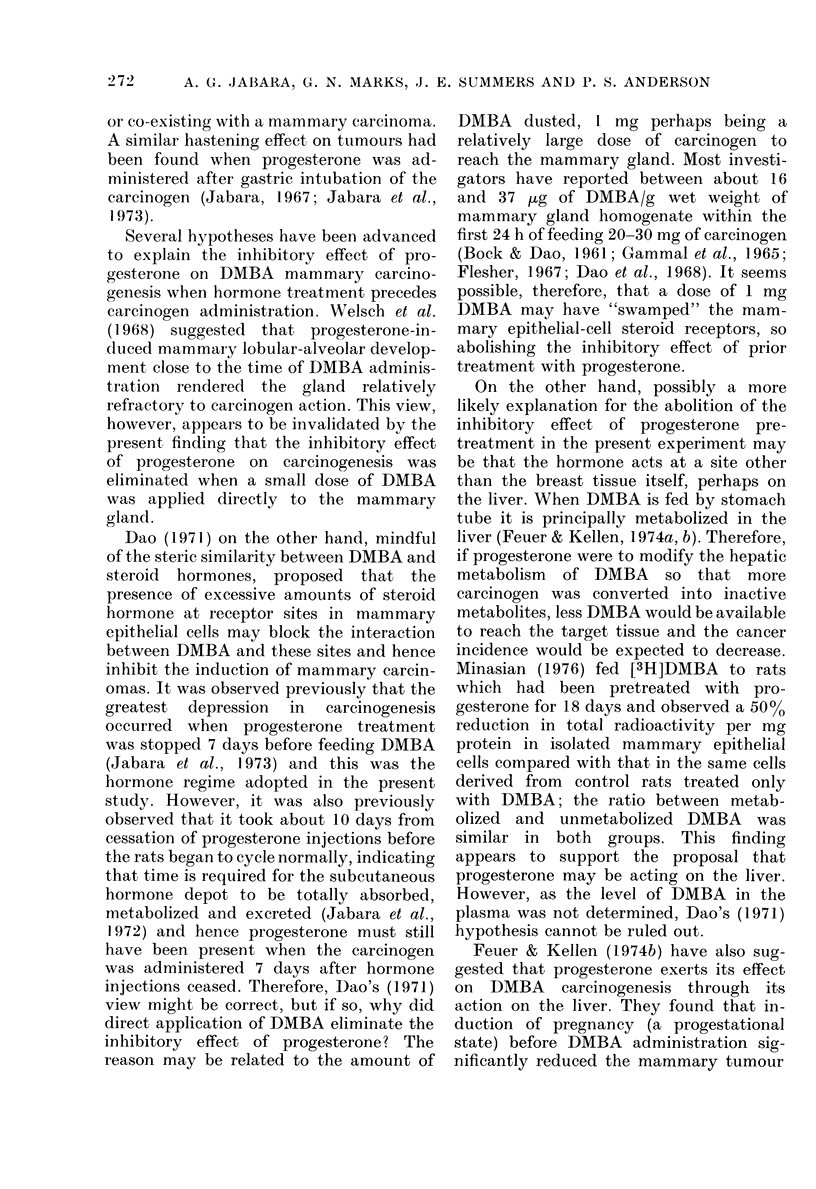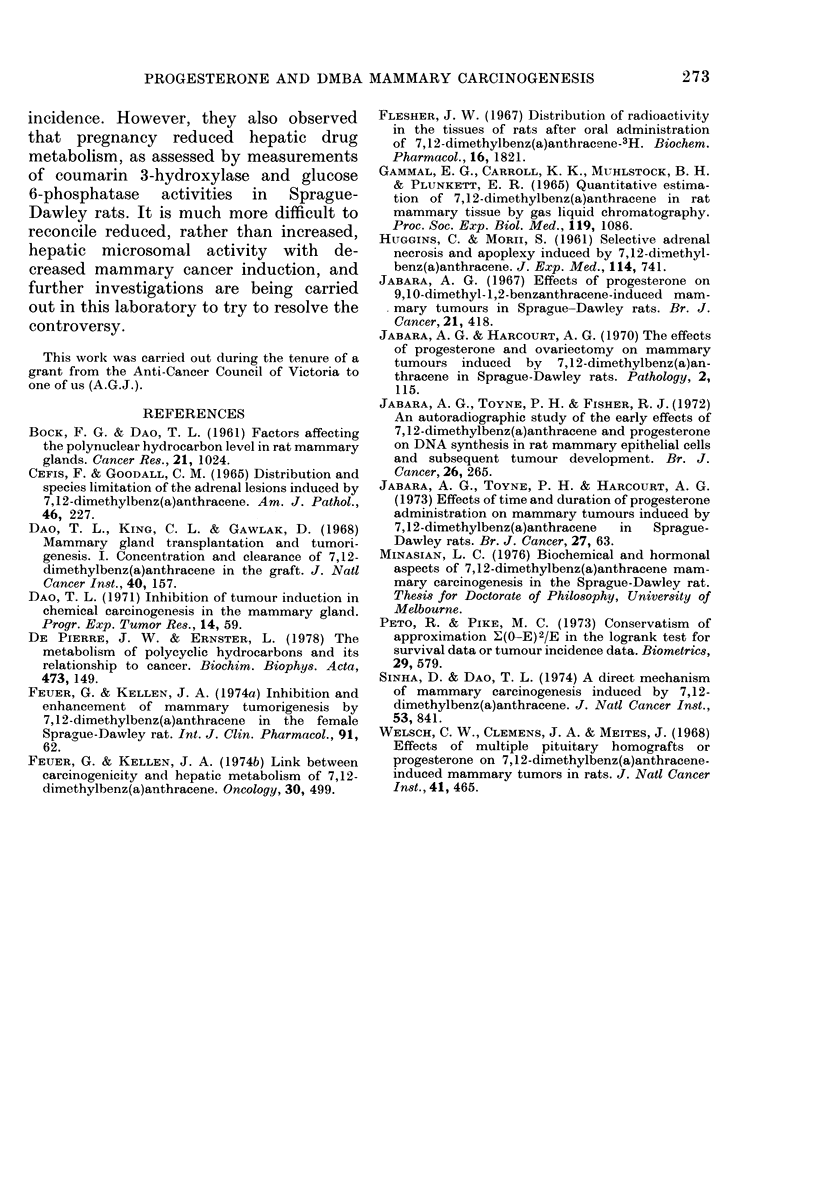# Effects of progesterone on mammary carcinogenesis by DMBA applied directly to rat mammae.

**DOI:** 10.1038/bjc.1979.175

**Published:** 1979-08

**Authors:** A. G. Jabara, G. N. Marks, J. E. Summers, P. S. Anderson

## Abstract

The effects and site(s) of action of progesterone on DMBA mammary carcinogenesis in the rat, when a small dose of the carcinogen was applied directly to the inguinal mammary gland, were investigated. No reduction in tumour yield was apparent when progesterone was administered s.c. for 18 days before dusting DMBA. This finding contrasts with a previously reported inhibitory effect on carcinogenesis when hormone treatment was followed by intragastric administration of DMBA. When progesterone injections were begun either 2 days before or 2 days after direct application of DMBA, and were continued until the end of the experiment (135 or 195 days) an enhancement in carcinogenesis was observed similar to that previously demonstrated after gastric intubation of DMBA. These findings, together with previously reported observations, suggest that progesterone may exert its inhibitory effect on carcinogenesis by acting at a site outside the breast, perhaps on the liver. However, it is likely that the hormone acts directly on the mammary tissue to exert its enhancing effect on tumorigenesis.


*Br. J. Cancer* (1979) **40**, 268

### EFFECTS OF PROGESTERONE ON MAMMARY CARCINOGENESIS BY DMBA APPLIED DIRECTLY TO RAT MAMMAE

A. G. JABARA*†, G. N. MARKS†, J. E. SUMMERS† AND P. S. ANDERSON‡

*From the Departments of Veterinary Paraclinical Sciences* and Pathology†, University of Melbourne, Parkville, Victoria 3052 and Department of Mathematics‡, Monash University, Clayton, Victoria 3168, Australia*

Received 27 February 1979 Accepted 12 April 1979

**Summary.—The effects and site(s) of action of progesterone on DMBA mammary carcinogenesis in the rat, when a small dose of the carcinogen was applied directly to the inguinal mammary gland, were investigated.**

**No reduction in tumour yield was apparent when progesterone was administered s.c. for 18 days before dusting DMBA. This finding contrasts with a previously reported inhibitory effect on carcinogenesis when hormone treatment was followed by intragastric administration of DMBA.**

**When progesterone injections were begun either 2 days before or 2 days after direct application of DMBA, and were continued until the end of the experiment (135 or 195 days) an enhancement in carcinogenesis was observed similar to that previously demonstrated after gastric intubation of DMBA.**

**These findings, together with previously reported observations, suggest that progesterone may exert its inhibitory effect on carcinogenesis by acting at a site outside the breast, perhaps on the liver. However, it is likely that the hormone acts directly on the mammary tissue to exert its enhancing effect on tumorigenesis.**

PREVIOUS INVESTIGATIONS have shown that exogenous progesterone (not carcinogenic *per se*) significantly reduced the tumour yield in Sprague-Dawley female rats when the hormone was administered for 18 days beginning 25 days before feeding a single dose of 7,12-dimethylbenz(a) anthracene (Jabara *et al.*, 1973). In contrast, when daily progesterone injections were begun either 2 days before or 2 days after feeding DMBA and were continued until the end of the experiment (135 or 195 days), a significant enhancement in mammary carcinogenesis was obtained (Jabara & Harcourt, 1970; Jabara *et al.*, 1973).

No insight was gained from the above investigations as to the site(s) of action of these contrasting progesterone regimes, *i.e.* whether the hormone acted at the level of the mammary gland or elsewhere. The present experiments were designed to investigate this question.

### MATERIALS AND METHODS

One hundred and forty non-inbred Sprague-Dawley virgin female rats were divided randomly into 7 groups of 20 rats each (Table I). They were housed 5 animals/cage and fed commercial pellets and water *ad libitum.* At 50 days of age each rat in Groups 2–7 (Table I) had its surgically exposed right 4th mammary gland "dusted" with 2 mg of powder containing a DMBA-cholesterol mixture (1:1) using the technique described by Sinha & Dao (1974). The inguinal mammary glands in the 50-day-old control rats (Group 1) were dusted with 2 mg of cholesterol powder only. Each animal in Groups 3, 4, 6 and 7 also received s.c. injections of 3 mg of progesterone (Sigma Chemical Co., U.S.A.) dissolved in 0·1 ml of corn oil/day 3 times a week. In Groups 3 and 6 progesterone injections were begun 25 days before DMBA administration (*i.e.* on their 25th day of age) and were continued for 18 days (DMBA+P-25 to -7). In Group 4 hormone injections were begun 2 days after DMBA (*i.e.* on their

52nd day of age) and were continued to the end of the experiment, *i.e.* 135 days (DMBA+P+2 to +135), while in Group 7 progesterone injections commenced 2 days before dusting DMBA (*i.e.* on their 48th day of age) and were continued for 197 days (DMBA+P–2 to +195).

Beginning 4 weeks after DMBA administration, all rats were palpated weekly and any mammary tumour recorded, measured and graphed as described previously (Jabara, 1967). At autopsy, mammary neoplasms were removed, fixed in 10% buffered formalin and 5$\mu$m paraffin sections were stained with haematoxylin and eosin.

In the statistical analysis of the results, "tumour incidence" and "latent periods" were analysed by means of the log rank test (Peto & Pike, 1973) and the "tumour growth behaviour" data were analysed using standard 2 × 2 contingency tables.

### RESULTS

In Groups 1–4 only 4 rats (2 in Group 2 and 2 in Group 3) failed to survive to the end of the experiment (135 days after dusting DMBA), all 4 dying from pneumonia. In the longer-term groups (5–7), considerably fewer animals survived the total experimental period of 195 days post-DMBA dusting (Table I) mainly because many rats had to be killed earlier because their tumours became large, ulcerated and invaded the abdominal wall, and sometimes extensively infiltrated the abdominal contents as well, causing cachexia of the host. In addition, 2, 3 and 2 rats in Groups 5–7, respectively, died from pneumonia.

### *Tumour incidence and latent periods*

Tumours developed only in the inguinal mammary glands where the DMBA was applied. In the 3 longer-term groups (5–7) the majority of rats (29/51) which developed mammary carcinomas also developed "non-mammary" neoplasms of the skin or body wall at the site of dusting (Table II). For each of the rats developing both types of neoplasm, it was impossible to determine to which tumour type the observed latent period and growth behaviour related. The data from the remaining animals in Groups 5–7 were therefore of little statistical use because of the

TABLE I.—*Effects of progesterone (P) on mammary tumour yield when the hormone was injected daily into rats either before or after dusting DMBA directly on the right 4th mammary gland*

| Group: | 1 | 2 | 3 | 4 | 5 | 6 | 7 |
|---|---|---|---|---|---|---|---|
| Treatment: | Cholesterol | DMBA | DMBA + P – 25 to – 7 | DMBA + P + 2 to + 135 | DMBA | DMBA + P – 25 to – 7 | DMBA + P – 2 to + 195 |
| Total rats | 20 | 20 | 17* | 20 | 20 | 20 | 20 |
| Survivors at 28 days | 20 | 20 | 17 | 20 | 20 | 20 | 20 |
| at 135 days | 20 | 18 | 15 | 20 | | | |
| at 195 days | | | | | 14 | 12 | 10 |
| No. of rats with mammary carcinomas | 0 | 12 | 12 | 15 | 17 | 18 | 16 |
| (%) | | (60) | (71) | (75) | (85) | (90) | (80) |
| Average latent period (days)† | 0 | 110 | 102 | 80 | 98 | 88 | 68 |
| (range) | | (77–126) | (89–118) | (63–104) | (61–149) | (36–155) | (40–112) |
| Growth behaviour of carcinomas | | | | | | | |
| No. growing continuously | 0 | 3 | 4 | 9 | 11 | 11 | 10 |
| No. remaining static | 0 | 9 | 8 | 3 | 1 | 2 | 0 |
| No. regressing | 0 | 0 | 0 | 0 | 0 | 0 | 0 |
| No. unclassified‡ | 0 | 0 | 0 | 3 | 5 | 5 | 6 |

* Twenty rats were allocated to this group, but 3 died before receiving DMBA and were therefore eliminated from all subsequent calculations and analyses.
† Average based on 10, 7, 9, 6, 7 and 6 rats in Groups 2–7, respectively.
‡ Classification was impossible as either no obvious distinction in size was apparent between the initial granuloma and the tumour which developed subsequently or, in the case of some combined neoplasms, it was impossible to determine whether growth was mainly related to the mammary neoplasm or to the tumour arising from the body wall or both.

TABLE II.—*Proportions of histological tumour types arising at the site of DMBA dusting*

| Group | Experiment duration (days) | Tumour type MC | MC + Non-MC | Non-MC | * |
|---|---|---|---|---|---|
| 2 | 135 | 11 | 1 | 2 | 0 |
| 3 | ,, | 12 | 0 | 0 | 0 |
| 4 | ,, | 12 | 3 | 2 | 0 |
| 5 | 195 | 7 | 10 | 2 | 1 |
| 6 | ,, | 9 | 9 | 0 | 0 |
| 7 | ,, | 6 | 10 | 2 | 0 |

MC = Mammary carcinoma.
Non-MC = Non-mammary tumour.
* Tumour classification impossible as the rat died and was cannibalized.

small number of mammary carcinomas which arose alone and the biases caused by the censoring. For this reason only trends will be reported for these 3 groups.

In Groups 1–4, the shorter duration produced a greatly decreased incidence of "non-mammary" tumours, and of the 39 rats which developed mammary carcinomas (Groups 2–4) only 4 developed neoplasms of the body wall or skin as well (Table II). These 4 animals were eliminated from the statistical analysis of latent period.

When the rats were first palpated 4 weeks after dusting DMBA, a very small lump was palpable in the dusted gland in every animal except those in Group 1, which were dusted with cholesterol powder only. These lumps consisted of a granulomatous reaction, presumably due to the DMBA, as it did not occur when only cholesterol was present, and the tumour latent period was therefore measured from the time of dusting DMBA until the lump began to grow. However, in each of 1, 5 and 3 rats in Groups 2–4, respectively, the lump palpated at 4 weeks did not vary appreciably in size throughout the remainder of the experiment, though histological examination at autopsy revealed it to be a mammary adenocarcinoma. A latent period could not be determined in these 9 rats and therefore they were eliminated from the statistical analysis.

The dusting vehicle, cholesterol, was not carcinogenic *per se*, as shown by the absence of a tumour in all rats in Group 1 (Table I).

The mammary-cancer incidence and average tumour latent period in rats in Group 2, treated only with DMBA, were not significantly different from those in Group 3 which had also received progesterone before the carcinogen (Table I). However, in Group 4, where progesterone was administered *after* DMBA, the log rank test showed a significantly higher incidence of mammary carcinomas and shorter latent period than in Groups 2 and 3 combined ($P < 0{\cdot}05$) (Table I).

In the longer-term groups (5–7) it was not possible to test for differences in tumour incidence or length of latent period for the reasons outlined above. There appeared, however, to be no substantial difference between the mammary-cancer incidences in the 3 groups (Table I). As seen in the shorter-term group (4), progesterone treatment after DMBA appeared to shorten markedly the average tumour latent period (Group 7) compared with that in Groups 5 or 6, whilst progesterone treatment before DMBA dusting (Group 6) produced the same induction time as in the controls (Group 5) (Table I).

*Tumour growth behaviour and tumour size*

Preliminary examination of the data relating to the growth behaviour of the mammary carcinomas which arose in Groups 2–4 suggested that progesterone treatment *before* DMBA dusting (Group 3) made no difference to neoplastic growth behaviour compared with that in the controls (Group 2), whilst progesterone treatment *after* DMBA appeared to stimulate many more carcinomas to grow continuously. This effect is highly significant statistically since, after pooling Groups 2 and 3 and neglecting the 3 unclassified carcinomas in Group 4, the standard $2 \times 2$ contingency table gave a $\chi^2$ of 6·81 which is significant at the 1% level.

Whilst tumour size is related to tumour growth behaviour, it is noteworthy that at autopsy 53% of the neoplasms in Group 4 (8/15) had obtained an average size of 2 cm or larger, compared with only 25% in Group 3 (3/12) and 8% in Group 2 (1/12).

*Types of tumours*

All mammary carcinomas which arose in Groups 2–7 (Table I) were adenocarcinomas, mainly of the papillary cystic variety (Jabara, 1967) and progesterone did not seem to influence either their macroscopic or microscopic appearances, regardless of whether the hormone was administered before or after DMBA dusting.

One rat in Group 4, in addition to the 12 animals bearing malignant mammary growths, developed a benign mammary adenoma (Jabara, 1967) in the dusted gland, while in the longer-term groups, a fibroadenoma co-existing with a mammary adenocarcinoma was seen in 1 rat in Group 5, 2 in Group 6 and 3 in Group 7. In addition, 2 rats in Group 7 developed only a fibroadenoma in the dusted gland.

Non-mammary neoplasms derived from the skin or body wall also developed at the site of dusting in some rats (Table II). These tumours sometimes arose alone, but more commonly co-existed with mammary growths (Table II). Histologically, most of these other tumours were fibrosarcomas, a few were rhabdomyosarcomas or mixed-cell sarcomas and one was a squamous-cell carcinoma.

### DISCUSSION

From recent studies it appears that DMBA requires metabolic activation to its carcinogenic form (Feuer & Kellen, 1974*a*, *b*; De Pierre & Ernster, 1978). Hence, induction of breast carcinomas after direct application of DMBA to the mammary gland indicates that the breast tissue contains the necessary enzymes to activate DMBA, as direct application of such a small dose (1 mg) of carcinogen appears to eliminate any systemic effect. This was evidenced by the fact that tumours arose only in the mammary gland that had been dusted with carcinogen and, further, that no rat died from adrenal apoplexy and the adrenal glands removed at autopsy showed no histological evidence of cortical damage, a common finding after intragastric administration of DMBA (Huggins & Morii, 1961; Cefis & Goodall, 1965).

The present experiment also showed that the inhibitory effect of progesterone pretreatment on DMBA mammary carcinogenesis (Jabara *et al.*, 1973) is abolished when the carcinogen is applied directly to the mammary gland, whether the animals survived for 135 or 195 days. On the other hand, an enhancing effect of progesterone on DMBA mammary carcinogenesis was still found after direct application of the carcinogen, similar to that previously found after gastric intubation of DMBA (Jabara, 1967; Jabara *et al.*, 1973).

These differing effects of progesterone on DMBA carcinogenesis suggest that the hormone may be acting at two distinct sites. In the case of carcinogenic enhancement by progesterone, it is suggested that the hormone acts directly on the mammary gland to stimulate growth of pre-existing tumours or malignantly transformed cells. This would explain the greater number of continuously growing carcinomas in Groups 4 and 7 than in the controls, the fact that many more neoplasms attained a much larger size in these 2 groups, and the observation that the appearance of benign mammary tumours, as well as malignant ones, tended to be hastened by post-DMBA treatment with progesterone. The last finding was especially obvious in the longer-term group (7), the benign tumours appearing either alone or co-existing with a mammary carcinoma. A similar hastening effect on tumours had been found when progesterone was administered after gastric intubation of the carcinogen (Jabara, 1967; Jabara *et al.*, 1973).

Several hypotheses have been advanced to explain the inhibitory effect of progesterone on DMBA mammary carcinogenesis when hormone treatment precedes carcinogen administration. Welsch *et al.* (1968) suggested that progesterone-induced mammary lobular-alveolar development close to the time of DMBA administration rendered the gland relatively refractory to carcinogen action. This view, however, appears to be invalidated by the present finding that the inhibitory effect of progesterone on carcinogenesis was eliminated when a small dose of DMBA was applied directly to the mammary gland.

Dao (1971) on the other hand, mindful of the steric similarity between DMBA and steroid hormones, proposed that the presence of excessive amounts of steroid hormone at receptor sites in mammary epithelial cells may block the interaction between DMBA and these sites and hence inhibit the induction of mammary carcinomas. It was observed previously that the greatest depression in carcinogenesis occurred when progesterone treatment was stopped 7 days before feeding DMBA (Jabara *et al.*, 1973) and this was the hormone regime adopted in the present study. However, it was also previously observed that it took about 10 days from cessation of progesterone injections before the rats began to cycle normally, indicating that time is required for the subcutaneous hormone depot to be totally absorbed, metabolized and excreted (Jabara *et al.*, 1972) and hence progesterone must still have been present when the carcinogen was administered 7 days after hormone injections ceased. Therefore, Dao's (1971) view might be correct, but if so, why did direct application of DMBA eliminate the inhibitory effect of progesterone? The reason may be related to the amount of DMBA dusted, 1 mg perhaps being a relatively large dose of carcinogen to reach the mammary gland. Most investigators have reported between about 16 and 37 $\mu$g of DMBA/g wet weight of mammary gland homogenate within the first 24 h of feeding 20–30 mg of carcinogen (Bock & Dao, 1961; Gammal *et al.*, 1965; Flesher, 1967; Dao *et al.*, 1968). It seems possible, therefore, that a dose of 1 mg DMBA may have "swamped" the mammary epithelial-cell steroid receptors, so abolishing the inhibitory effect of prior treatment with progesterone.

On the other hand, possibly a more likely explanation for the abolition of the inhibitory effect of progesterone pretreatment in the present experiment may be that the hormone acts at a site other than the breast tissue itself, perhaps on the liver. When DMBA is fed by stomach tube it is principally metabolized in the liver (Feuer & Kellen, 1974*a*, *b*). Therefore, if progesterone were to modify the hepatic metabolism of DMBA so that more carcinogen was converted into inactive metabolites, less DMBA would be available to reach the target tissue and the cancer incidence would be expected to decrease. Minasian (1976) fed [$^3$H]DMBA to rats which had been pretreated with progesterone for 18 days and observed a 50% reduction in total radioactivity per mg protein in isolated mammary epithelial cells compared with that in the same cells derived from control rats treated only with DMBA; the ratio between metabolized and unmetabolized DMBA was similar in both groups. This finding appears to support the proposal that progesterone may be acting on the liver. However, as the level of DMBA in the plasma was not determined, Dao's (1971) hypothesis cannot be ruled out.

Feuer & Kellen (1974*b*) have also suggested that progesterone exerts its effect on DMBA carcinogenesis through its action on the liver. They found that induction of pregnancy (a progestational state) before DMBA administration significantly reduced the mammary tumour incidence. However, they also observed that pregnancy reduced hepatic drug metabolism, as assessed by measurements of coumarin 3-hydroxylase and glucose 6-phosphatase activities in Sprague-Dawley rats. It is much more difficult to reconcile reduced, rather than increased, hepatic microsomal activity with decreased mammary cancer induction, and further investigations are being carried out in this laboratory to try to resolve the controversy.

This work was carried out during the tenure of a grant from the Anti-Cancer Council of Victoria to one of us (A.G.J.).

### REFERENCES

BOCK, F. G. & DAO, T. L. (1961) Factors affecting the polynuclear hydrocarbon level in rat mammary glands. *Cancer Res.*, **21**, 1024.

CEFIS, F. & GOODALL, C. M. (1965) Distribution and species limitation of the adrenal lesions induced by 7,12-dimethylbenz(a)anthracene. *Am. J. Pathol.*, **46**, 227.

DAO, T. L., KING, C. L. & GAWLAK, D. (1968) Mammary gland transplantation and tumorigenesis. I. Concentration and clearance of 7,12-dimethylbenz(a)anthracene in the graft. *J. Natl Cancer Inst.*, **40**, 157.

DAO, T. L. (1971) Inhibition of tumour induction in chemical carcinogenesis in the mammary gland. *Progr. Exp. Tumor Res.*, **14**, 59.

DE PIERRE, J. W. & ERNSTER, L. (1978) The metabolism of polycyclic hydrocarbons and its relationship to cancer. *Biochim. Biophys. Acta*, **473**, 149.

FEUER, G. & KELLEN, J. A. (1974*a*) Inhibition and enhancement of mammary tumorigenesis by 7,12-dimethylbenz(a)anthracene in the female Sprague-Dawley rat. *Int. J. Clin. Pharmacol.*, **91**, 62.

FEUER, G. & KELLEN, J. A. (1974*b*) Link between carcinogenicity and hepatic metabolism of 7,12-dimethylbenz(a)anthracene. *Oncology*, **30**, 499.

FLESHER, J. W. (1967) Distribution of radioactivity in the tissues of rats after oral administration of 7,12-dimethylbenz(a)anthracene-$^{3}$H. *Biochem. Pharmacol.*, **16**, 1821.

GAMMAL, E. G., CARROLL, K. K., MUHLSTOCK, B. H. & PLUNKETT, E. R. (1965) Quantitative estimation of 7,12-dimethylbenz(a)anthracene in rat mammary tissue by gas liquid chromatography. *Proc. Soc. Exp. Biol. Med.*, **119**, 1086.

HUGGINS, C. & MORII, S. (1961) Selective adrenal necrosis and apoplexy induced by 7,12-dimethylbenz(a)anthracene. *J. Exp. Med.*, **114**, 741.

JABARA, A. G. (1967) Effects of progesterone on 9,10-dimethyl-1,2-benzanthracene-induced mammary tumours in Sprague–Dawley rats. *Br. J. Cancer*, **21**, 418.

JABARA, A. G. & HARCOURT, A. G. (1970) The effects of progesterone and ovariectomy on mammary tumours induced by 7,12-dimethylbenz(a)anthracene in Sprague-Dawley rats. *Pathology*, **2**, 115.

JABARA, A. G., TOYNE, P. H. & FISHER, R. J. (1972) An autoradiographic study of the early effects of 7,12-dimethylbenz(a)anthracene and progesterone on DNA synthesis in rat mammary epithelial cells and subsequent tumour development. *Br. J. Cancer*, **26**, 265.

JABARA, A. G., TOYNE, P. H. & HARCOURT, A. G. (1973) Effects of time and duration of progesterone administration on mammary tumours induced by 7,12-dimethylbenz(a)anthracene in Sprague-Dawley rats. *Br. J. Cancer*, **27**, 63.

MINASIAN, L. C. (1976) Biochemical and hormonal aspects of 7,12-dimethylbenz(a)anthracene mammary carcinogenesis in the Sprague-Dawley rat. *Thesis for Doctorate of Philosophy, University of Melbourne.*

PETO, R. & PIKE, M. C. (1973) Conservatism of approximation $\Sigma(0-E)^2/E$ in the logrank test for survival data or tumour incidence data. *Biometrics*, **29**, 579.

SINHA, D. & DAO, T. L. (1974) A direct mechanism of mammary carcinogenesis induced by 7,12-dimethylbenz(a)anthracene. *J. Natl Cancer Inst.*, **53**, 841.

WELSCH, C. W., CLEMENS, J. A. & MEITES, J. (1968) Effects of multiple pituitary homografts or progesterone on 7,12-dimethylbenz(a)anthracene-induced mammary tumors in rats. *J. Natl Cancer Inst.*, **41**, 465.